# 2231 Research partnership, community commitment, and the people-to-people for Puerto Rico (#p2p4PUR) Movement: Researchers and citizens in solidarity

**DOI:** 10.1017/cts.2018.262

**Published:** 2018-11-21

**Authors:** Jose G. Perez-Ramos, Hector T. Zayas, Nancy R. Cardona Cordero, Dulce M. Del Rio Pineda, Colleen Murphy, Carmen M. Velez Vega, Timothy De Ver Dye

**Affiliations:** 1 University of Rochester Medical Center; 2 University of Puerto Rico, Medical Science Campus; 3 Mujeres De Isla, Inc

## Abstract

OBJECTIVES/SPECIFIC AIMS: Island communities face greater environmental risks creating challenges in their populations. A community and participatory qualitative research method aiming to understand community perspectives regarding the ecology and environmental risks of the island of Culebra was performed to develop a community-centered Information and Communications Technology (ICT) intervention (an app). The island of Culebra, a municipality from the archipelago of Puerto Rico is located 17 miles from the eastern coast of Puerto Rico’s main island. This ICT—termed mZAP (Zonas, Acción & Protección)—is part of a Translational Biomedical doctoral degree dissertation housed at the University of Rochester’s Clinical Translational Science Institute (CTSI) Informatics Core funded by an NIH Clinical Translational Science Award (CTSA). In September 2017, the island of Culebra faced 2 major category hurricanes 2 weeks apart. Hurricane Irma and Hurricane Maria devastated homes, schools, health clinics, and local businesses, disrupting an already-fragile ecological balance on the island. METHODS/STUDY POPULATION: These 2 storms catastrophically affected the archipelago of Puerto Rico. Culebra’s geographically isolated location, along with the inefficient response from authorities, exacerbated the stressors caused by these natural disasters, increasing the gap of social determinants of health, including the lack of potable water. Leveraging a community engagement partnership established before the hurricanes by the mZAP participatory research, which naturally halted once the hurricanes hit a new humanitarian objective formed to deliver aid. Along with another NIH funded RCMI Translational Research Network, or RTRN institution (University of Puerto Rico, Medical Science Campus) students and faculty, The Puerto Rico Testsite for Exploring Contamination Threats Program (PROTECT) an NIEHS Funded Grant, and the National Guard, a “people to people” approach was established to ascertain needs and an opportunity to meet those needs. A people-to-people approach brings humanitarian needs, identified directly by the community to the people who need it most; without intermediaries and bureaucratic delays that typically occur during catastrophes. RESULTS/ANTICIPATED RESULTS: The consumption of potable water in plastic bottles and subsequent accumulation of plastic material has proven to be collateral damage of a vulnerable water distribution system creating another environmental hazard on the island of Culebra. Therefore, this humanitarian partnership, worked to delivered community and family sized water filters, providing a safe environmental alternative to drinkable water for the island. The success of this approach, People to People for Puerto Rico (#p2p4PUR), demonstrated the power of genuine community engagement—arising from a previous clinical research partnership—and true established commitment with members of the community. DISCUSSION/SIGNIFICANCE OF IMPACT: Research partnerships can (and should, when needed) lead to humanitarian partnerships that extend beyond research objectives. Research may subsequently be adapted based on new realities associated with natural disasters and the altered nature of existing partnerships, allowing for a rapid response to communities need. Further, #p2p4PUR was not only able to channel a partnership humanitarian response but also created an opportunity to reflect on how the commitment between members of society and academia (researchers) can create beneficial bilateral relationships, always putting the community needs first. The resulting shared experience elevates community interest and engagement with researchers, and helps researchers see communities as true partners, rather than—simply—research subjects.

